# Expression and Role of Voltage-Gated Sodium Channels in Human Dorsal Root Ganglion Neurons with Special Focus on Nav1.7, Species Differences, and Regulation by Paclitaxel

**DOI:** 10.1007/s12264-017-0132-3

**Published:** 2017-04-19

**Authors:** Wonseok Chang, Temugin Berta, Yong Ho Kim, Sanghoon Lee, Seok-Yong Lee, Ru-Rong Ji

**Affiliations:** 1 Department of Anesthesiology, Duke University Medical Center, Durham, NC 27710 USA; 2 Department of Physiology and Biophysics, College of Medicine, Eulji University, Daejeon, Korea; 3 Pain Research Center, Department of Anesthesiology, University of Cincinnati Medical Center, Cincinnati, OH 45267 USA; 4 Department of Physiology, College of Medicine, Gachon University, Incheon, Korea; 5 Department of Biochemistry, Duke University Medical Center, Durham, NC 27710 USA; 6 Department of Neurobiology, Duke University Medical Center, Durham, NC 27710 USA

**Keywords:** Dorsal root ganglion, Neuropathic pain, Paclitaxel, Voltage-gated sodium channels

## Abstract

Voltage-gated sodium channels (Navs) play an important role in human pain sensation. However, the expression and role of Nav subtypes in native human sensory neurons are unclear. To address this issue, we obtained human dorsal root ganglion (hDRG) tissues from healthy donors. PCR analysis of seven DRG-expressed Nav subtypes revealed that the hDRG has higher expression of Nav1.7 (~50% of total Nav expression) and lower expression of Nav1.8 (~12%), whereas the mouse DRG has higher expression of Nav1.8 (~45%) and lower expression of Nav1.7 (~18%). To mimic Nav regulation in chronic pain, we treated hDRG neurons in primary cultures with paclitaxel (0.1–1 μmol/L) for 24 h. Paclitaxel increased the Nav1.7 but not Nav1.8 expression and also increased the transient Na^+^ currents and action potential firing frequency in small-diameter (<50 μm) hDRG neurons. Thus, the hDRG provides a translational model in which to study “human pain in a dish” and test new pain therapeutics.

## Introduction

Over the last 20 years, great progress has been made in identifying novel pain targets and illustrating the neuronal, immune, and glial mechanisms of pathological pain in animal models [1–4]. Despite the progress in preclinical studies and substantial investment by the pharmaceutical industry, little progress has been made in developing novel, efficacious, and safe analgesics for human pain conditions [5, 6]. A translational gap from rodents to humans has been blamed for the failure in developing pain medications [5, 6]. To address this issue, several groups including our own have begun to investigate pain mechanisms in native primary sensory neurons in human dorsal root ganglia (hDRGs) obtained from donors [7–11]. Davidson *et al.* demonstrated that a high proportion of hDRG neurons respond to the algogens allyl isothiocyanate and ATP, as well as the pruritogens histamine and chloroquine [7]. Importantly, hDRG neurons also respond to the inflammatory mediators bradykinin and prostaglandin E2, showing evidence of peripheral sensitization [7].

Voltage-gated sodium (Nav) channels play a critical role in the initiation and propagation of action potentials in excitable cells such as neurons and are the major targets of analgesics [12–14]. Nine subtypes of Nav (Nav1.1–Nav1.9) have been identified; each subtype plays a distinct physiological role and also exhibits distinct expression patterns in DRG neurons. For example, Nav1.7–Nav1.9 are mainly expressed in small nociceptive neurons in DRGs [12, 15–19]. In particular, recent genetic studies have demonstrated a critical role of Nav1.7 in pain sensation in humans. Loss-of-function mutations in *SCN9A,* the gene that codes for Nav1.7 in humans, result in a congenital inability to sense pain and anosmia without affecting other sensations such as touch and temperature [20, 21]. Conversely, gain-of-function mutations lead to episodic pain such as primary erythromelalgia and paroxysmal extreme pain disorder [22, 23]. Despite an essential role of Navs in human pain regulation, little is known about their expression and function in native human sensory neurons. Recently, Han *et al.* showed that human Nav1.8 channels display slower inactivation kinetics and produce larger persistent current and ramp current than previously reported in other species [8]. In this study, we set out to compare the relative expression levels of different Nav subtypes in mouse DRG (mDRG) and hDRG tissues. We found that hDRG has a high ratio of Nav1.7 expression and low ratio of Nav1.8 expression compared to mDRG. To recapitulate chronic pain-induced changes in sodium channel expression, we treated dissociated hDRG neurons with paclitaxel, a chemotherapy agent that induces neuropathic pain [10]. Our data demonstrated that paclitaxel not only increased the expression of Nav1.7 but also enhanced the function of Nav1.7, as revealed by enlarged amplitude of sodium currents and increased firing frequency of action potentials, in human nociceptive neurons.

## Materials and Methods

### Reagents

Paclitaxel was purchased from Sigma (St. Louis, MO). Specific primers were designed using the Real-Time PCR web tool from IDT SciTools (Integrated DNA Technologies, Coralville, IA).

### Animals

Adult CD1 mice (8–10 weeks old, both sexes) were obtained from Charles River Laboratories (Wilmington, MA) and used for PCR analysis. All the animal procedures performed in this study were approved by the Animal Care Committee of Duke University Medical Centre.

### Human DRG Tissues

Human DRGs were obtained from healthy donors through the National Disease Research Interchange (Philadelphia, PA) with permission from the Duke University Institutional Review Board. Postmortem L3–L5 DRGs were dissected from donors (see Table 1 for further details) and delivered in ice-cold culture medium to the laboratory at Duke University within 24–72 h of the donor’s death.

**Table 1:** Sex, age, and ethnicity of human DRG donors.

No.	Sex	Age (years)	Ethnicity
1	Female	76	Caucasian
2	Male	67	Caucasian
3	Female	66	Caucasian
4	Male	66	Caucasian
5	Female	58	African-American
6	Female	55	Caucasian
7	Male	54	Caucasian
8	Female	50	African-American
9	Male	49	Caucasian
10	Male	31	Caucasian
11	Male	29	Caucasian
12	Male	27	Caucasian
13	Male	55	Caucasian
14	Male	41	Caucasian
15	Female	27	Caucasian

### Immunohistochemistry

Human DRGs were fixed in 4% paraformaldehyde overnight and then sections (12 μm) were cut on a cryostat. The sections were first blocked with 2% BSA for 1 h at room temperature, then incubated with peripherin (1:500, rabbit, Sigma) or a mixture of primary polyclonal TRPV1 antibody (1:400, rabbit, Neuromics, Edina, MN) and monoclonal NF200H antibody (1:1000, mouse, Sigma) overnight at 4 °C. The sections were then incubated for 2 h at room temperature with cyanine 3 (Cy3)- and FITC-conjugated secondary antibodies (1:400; Jackson ImmunoResearch, West Grove, PA). The DRG sections were then stained with DAPI (1:1000, Sigma, for 5 min) and examined under a Nikon fluorescence microscope. Images were captured with a CCD Spot camera and analyzed with NIH Image J software or Adobe PhotoShop.

### Quantitative Real-Time RT-PCR

DRG tissues were rapidly isolated in RNAse-free conditions. Total RNAs were extracted using an RNeasy Plus Mini kit (Qiagen, Germantown, MD). The quantity and quality of the eluted RNA samples were verified using a NanoDrop spectrophotometer (Thermo Fisher Scientific, Waltham, MA). Total RNAs (0.5 μg) were reverse-transcribed using the QuantiTect Reverse Transcription Kit according to the protocol of the manufacturer (Qiagen). The sequences of human Nav primers are listed in Table 2 (the sequences of mouse Nav primers were obtained from a previous publication [24]). We performed gene-specific mRNA analyses using the Bio-Rad CFX96 Real-Time RT-PCR system (BioRad, Hercules, CA). Quantitative PCR amplification reactions contained the same amount of reverse transcription product: 7.5 μL of Kapa Sybr® Fast Bio-Rad iCycler 2X qPCR Master Mix (Kapa Biosystems, Wilmington, MA) and 200 nmol/L forward and reverse primers in a final volume of 15 μL. The thermal cycling conditions were: 3 min of polymerase activation at 95 °C, 45 cycles of denaturation at 95 °C for 10 s, and annealing and extension at 60 °C for 30 s, followed by a DNA melting curve for the determination of amplicon specificity. The expression level of the target mRNA was normalized to that of GAPDH mRNA and analyzed using the standard 2^−ΔΔCT^ method. The relative mRNA expression of each Nav subtype is shown as a percentage of the total Navs (all seven subtypes) tested in DRGs [24].

**Table 2:** Sequences of PCR primers for seven human Nav subtypes.

Gene	Forward	Reverse	Amplicon size	Accession number
Nav1.1	TCTCTTGCGGCTATTGAAAGAC	GGGCCATTTTCGTCGTCATCT	86	NM_001202435
Nav1.2	ATAGCGCTGTGGACTGCAATG	CTGTTTCAGTAGTTGTGCCCTCTG	102	NM_001040143
Nav1.3	CACCTTTAGCTGGGCTTTCCTG	CCAGCAGCACGTAATGTCAAC	90	NM_001081676
Nav1.6	CTGCGATCTTTCCGATTGCTC	AGGGCACCCACTGAATTTCC	98	NM_014191
Nav1.7	GGTTTCAGCACAGATTCAGGTC	CCAGCTGAAAGTGTCAAAGCTC	102	NM_002977
Nav1.8	CATCAAAGTGTCTGTCCACTCG	TTTCTCTGGAAGGTCAGTTCGG	97	NM_006514
Nav1.9	GGCACCGTTATCATCAACTGC	AAATCCCAGTGAAGACACACTC	100	NM_014139
GAPDH	AGCCACATCGCTCAGACAC	GCCCAATACGACCAAATCC	66	NM_002046

### Human DRG Neuron Cultures

Upon delivery, DRGs were rapidly dissected from the nerve roots, minced in Ca^2+^-free Hank’s balanced salt solution (Thermo Fisher Scientific), and cultures were prepared as previously reported [11]. DRGs were digested at 37 °C in a humidified CO_2_ incubator for 120 min with collagenase Type II (Worthington, Lakewood, NJ; 290 units/mg, 12 mg/mL final concentration) and dispase II (Roche, Basel, Switzerland; 1 unit/mg, 20 mg/mL) in phosphate-buffered saline with 10 mmol/L HEPES, pH adjusted to 7.4 with NaOH. hDRGs were mechanically dissociated using fire-polished pipettes, filtered through a 100-μm nylon mesh, and centrifuged for 5 min (500 g). The DRG cell pellet was re-suspended and plated on 0.5 mg/mL poly-D-lysine-coated glass coverslips. The DRG cultures were grown in Neurobasal medium supplemented with 10% fetal bovine serum, 2% B-27 supplement, and 1% penicillin/streptomycin.

### Whole-Cell Patch-Clamp Recordings in Dissociated Human DRG Neurons

Whole-cell patch-clamp recordings in small-diameter (<50 μm) hDRG neurons were conducted at room temperature. We used patch pipettes to record transient Na^+^ currents and action potentials with an EPC10 amplifier (HEKA, Holliston, MA) and an Axopatch-200B amplifier with a Digidata 1440A (Axon Instruments, Sunnyvale CA). The patch pipettes were pulled from borosilicate capillaries (World Precision Instruments, Inc., Sarasota, FL). The resistance was 3–4 MΩ when filled with pipette solution. The recording chamber (300 µL) was continuously superfused at 3–4 mL/min. Series resistance was compensated (>80%) and leak subtraction was performed. Data were low-pass-filtered at 2 kHz and sampled at 10 kHz. Patchmaster (HEKA) and pClamp10 (Axon Instruments) softwares were used during experiments and analysis. For recording Na^+^ currents, the pipette solution contained (in mmol/L): CsCl 130, NaCl 9, MgCl_2_ 1, EGTA 10, HEPES 10, adjusted to pH 7.3 with CsOH. For action potential recordings, the pipette solution contained (in mmol/L): K-gluconate 136, NaCl 10, MgCl_2_ 1, EGTA 10, HEPES 10, Mg-ATP 2, adjusted to pH 7.3 with KOH. According to our preliminary study in hDRG neurons, action potentials were induced by 500 pA current injection for 1 s. The external solution for action potential recordings was composed of (in mmol/L): NaCl 140, KCl 5, CaCl_2_ 2, MgCl_2_ 1, HEPES 10, glucose 10, adjusted to pH 7.3 with NaOH. The external solution for recording transient Na^+^ currents contained (in mmol/L): NaCl 131, TEACl 10, CsCl 10, CaCl_2_ 1, MgCl_2_ 2, CdCl_2_ 0.3, 4-aminopyridine 3, HEPES 10, glucose 10, adjusted to pH 7.4 with NaOH. In voltage-clamp experiments, transient Na^+^ currents were evoked by a test pulse to 0 mV from the holding potential (−70 mV).

### Statistical Analysis

All data are expressed as mean ± SEM. Biochemical and electrophysiological data were tested using one-way or two-way ANOVA followed by the *post-hoc* Bonferroni test or unpaired two-tailed Student’s *t*-test. Pearson’s correlation was used to test the linear dependence of two variables. Statistical analyses were performed with Prism 6.0 (GraphPad Software) and the criterion for statistical significance was *P* < 0.05.

## Results

### Immunohistochemical Characterization of Human DRG Neurons

**Fig. 1:**
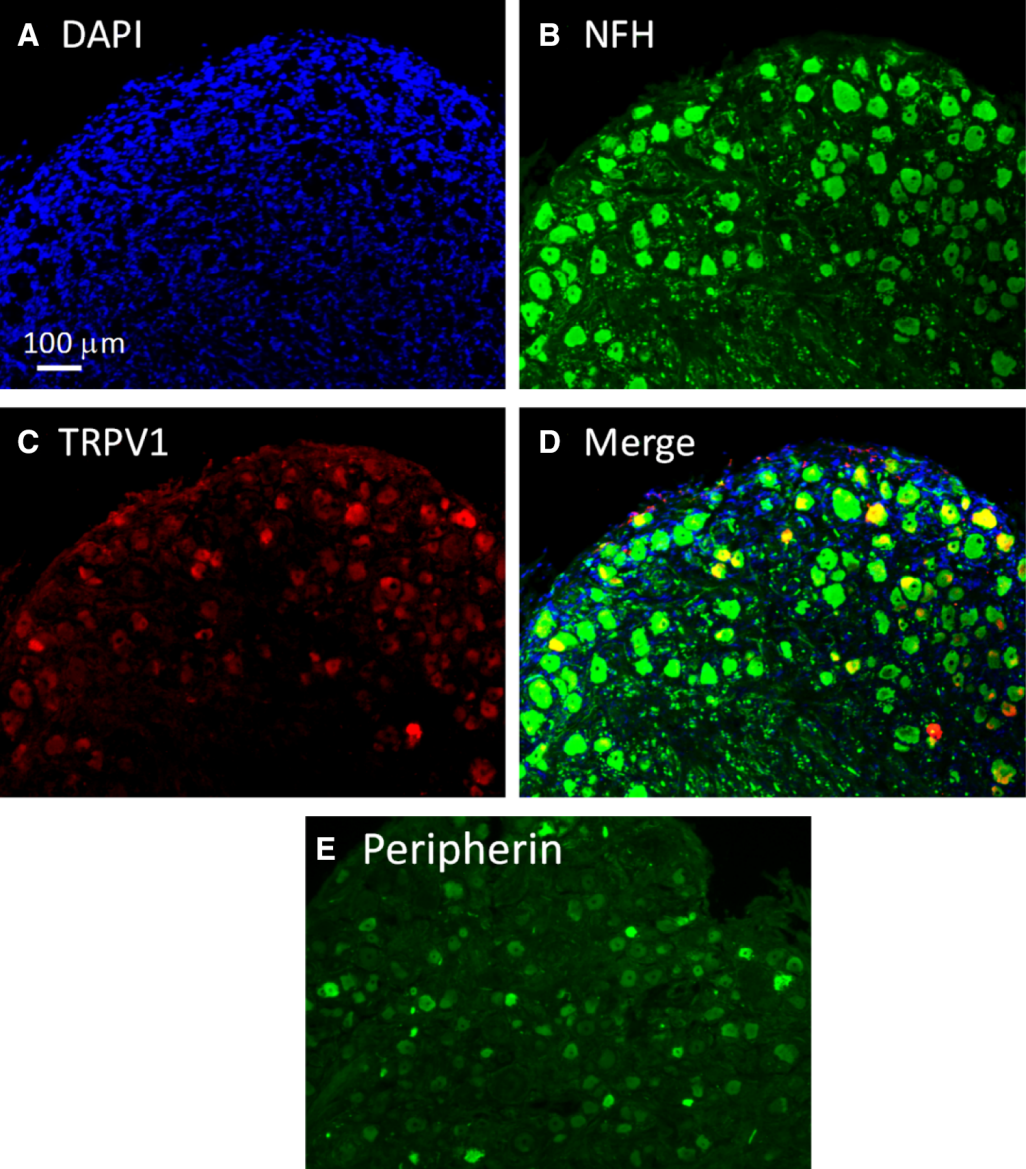
Histochemical characterization of human DRG neurons. **A** DAPI nuclear staining in an intact hDRG section. **B**, **C** Double-staining of neurofilament (NF200 or NFH, *green*, **B**) and TRPV1 (*red*, **C**) in the section. **D** Merged image of DAPI, NFH, and TRPV1 staining. **E** Immunostaining of peripherin in an hDRG section. *Scale bar*, 100 μm for **A**–**E**.

Immunofluorescent staining of hDRGs is a challenge, because of the high background of autofluorescence in human tissue [25]. We were able to obtain intact frozen sections of hDRGs, as demonstrated by DAPI staining, which shows all the nuclei in sections (Fig. 1A). Notably, hDRG neurons were much larger than rodent DRG neurons, and small hDRG neurons were identified with diameters of <50 μm [7, 11]. Neurofilament (NF200) is a marker for myelinated A-fiber neurons in rodent DRGs [10]. However, in hDRG sections, NF200 labeled both large and small neurons (Fig. 1B). Double-staining revealed co-localization of NF200 (NF200H) with TRPV1, a marker for C-fiber neurons in the rodent DRG [26] (Fig. 1C, D). Peripherin is another marker for small nociceptor neurons in the rodent DRG [27]. Consistently, peripherin was also expressed by small-diameter hDRG neurons (Fig. 1E). Limited size analysis showed that the average diameters of TRPV1- and peripherin-positive hDRG neurons were 44 ± 7 and 38 ± 7 μm, respectively (*n* = 28–37 neurons/group). Our results are in agreement with previous reports that hDRG neurons with diameters <50 μm can be regarded as small nociceptive neurons [7, 11].

### Distinct Expression of Sodium Channel Subtypes in Human and Mouse DRG Tissues

**Fig. 2:**
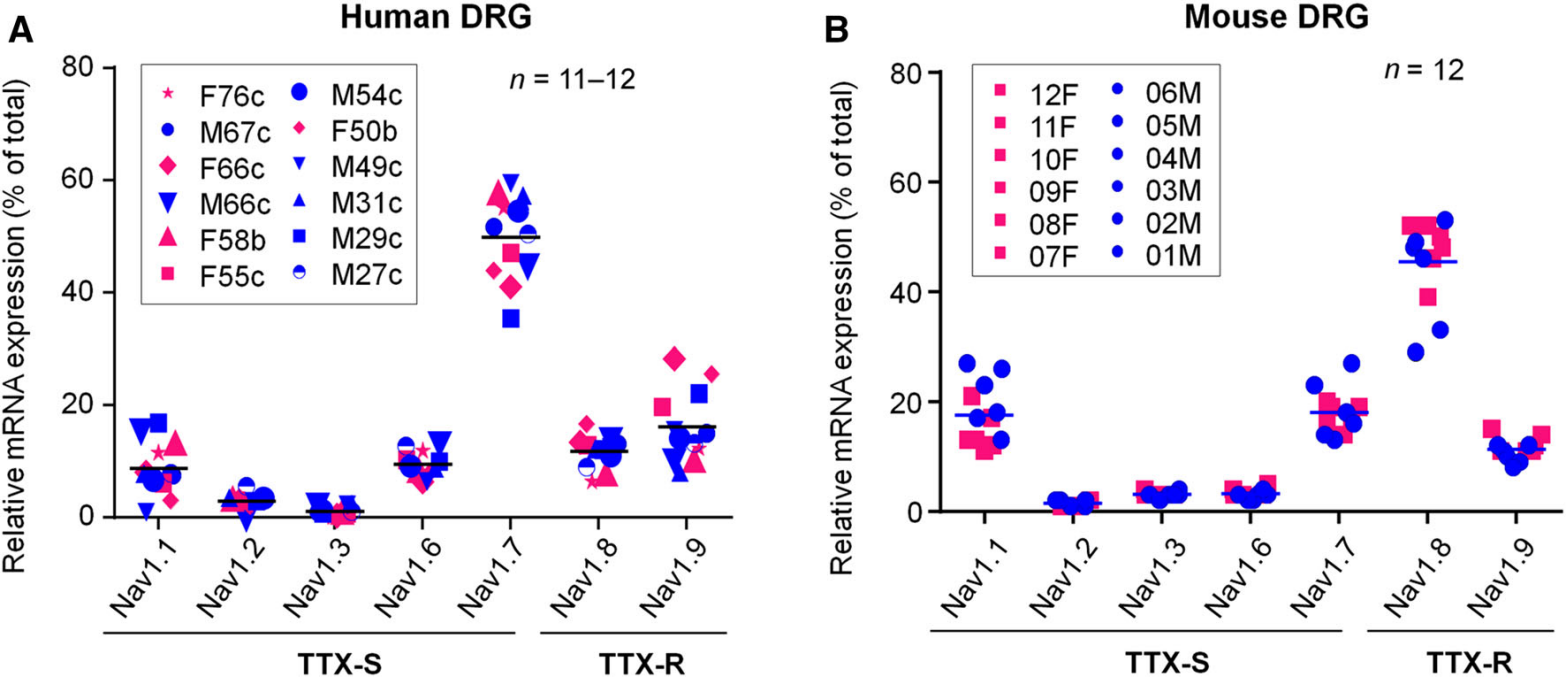
Distinct expression of sodium channel subtypes in human and mouse DRG tissues. **A, B** qPCR showing the relative percentage expression of TTX-sensitive Nav subtypes (TTX-S: Nav1.1, Nav1.2, Nav.1.3, Nav1.6, and Nav1.7) and TTX-resistant subtypes (TTX-R: Nav1.8 and Nav1.9) in hDRG (**A**) and mDRG (**B**) tissues. The percentage expression of each subtype is based on the total expression of all seven subtypes. Note the species differences in Nav1.6, Nav1.7, and Nav1.8 expression in hDRGs and mDRGs (*n* = 12 samples/group; blue, male samples; red, female samples; b, African_American; c, Caucasian; F, female; M, male.

We used quantitative real-time PCR (qPCR) to compare the transcriptional expression of seven Nav subtypes in hDRGs and mDRGs: TTX-sensitive Navs (Nav1.1, Nav1.2, Nav.1.3, Nav1.6, and Nav1.7) and TTX-resistant Navs (Nav1.8 and Nav1.9). Notably, all these subtypes are known to be expressed in rodent DRG neurons [15, 16, 28]. To define the relative expression levels of each subtype in mouse and human DRG tissues, we calculated the percentage expression of each subtype based on the total expression of all these subtypes (Fig. 2A,B). Striking species differences were found in Nav1.1, Nav1.6, Nav1.7, Nav1.8, and Nav1.9 (Table 3). The ratio of Nav1.7 expression in the hDRG (49.2 ± 5.6%) was much higher than that in the mDRG (18.2 ± 1.7%). On the other hand, the mDRG had a much higher ratio of Nav1.8 (44.9 ± 2.4%) than that of the hDRG (12.1 ± 2.1%). Also, the ratio of Nav1.6 was higher in the hDRG (8.8 ± 0.8%) than in the mDRG (3.3 ± 0.3%) (Fig. 2A, B).

**Table 3:** Statistics of species difference in the expression of mouse and human Nav1.1, Nav1.2, Nav1.3, Nav1.6, Nav1.7, Nav1.8, and Nav1.9 from the samples shown in Fig. 2.

Gene	Mean difference	95.00% CI of diff.	*P* value
Nav1.1	−8.85	−13.52 to −4.179	<0.0001
Nav1.2	1.411	−3.261 to 6.082	>0.9999
Nav1.3	−1.996	−6.667 to 2.676	>0.9999
Nav1.6	6.306	1.634 to 10.98	0.0022
Nav1.7	31.88	27.21 to 36.55	<0.0001
Nav1.8	−33.68	−38.35 to −29	<0.0001
Nav1.9	4.841	0.1695 to 9.512	0.0374

*N* = 12 samples/group; two-way ANOVA followed by *post-hoc* Bonferroni test.

### Sodium Channel Subtype Expression in Human DRGs Shows No Sex and Age Difference

**Fig. 3:**
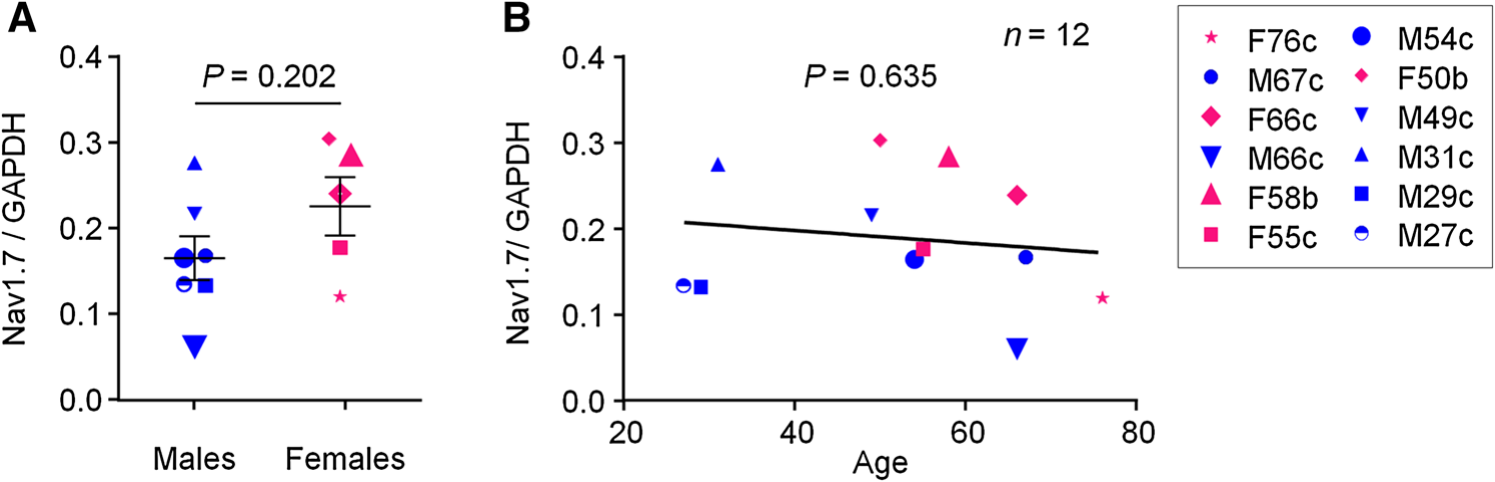
qPCR showing lack of sex and age differences in Nav 1.7 expression in human DRGs. **A** Comparison of Nav1.7 mRNA expression in male (*blue*) and female (*red*) hDRG tissues. Note the absence of a statistical difference (*P* = 0.202, unpaired Student’s *t*-test, *n* = 6 donors/group). **B** No correlation between age and Nav1.7 expression in hDRGs (*P* = 0.635, *n* = 12 donors, Pearson’s correlation). GAPDH was used as a positive control, and the value of Nav1.7 expression was normalized to that of GAPDH. All data are expressed as mean ± SEM.

We also investigated the sex differences in Nav subtype expression in hDRG and mDRG tissues. The relative expression of each Nav subtype in the hDRGs of 6 men and 6 women showed no sex difference in the expression of any subtype (Fig. 2A). Similarly, their relative expression in the mDRGs of 6 males and 6 females showed no sex difference in the expression of any mouse Nav subtype (Fig. 2B). Further comparison of the relative expression of Nav1.7 in hDRGs also revealed no statistical differences between men and women (Fig. 3A). We also analyzed Nav1.7 expression in hDRG donors of different ages, ranging from 27 to 76 years and found that although the expression was slightly lower in older donors, there was no significant correlation between Nav1.7 expression and age (Fig. 3B).

### Paclitaxel Increases Nav1.7 Expression, Transient Sodium Currents, and Action Potential Firing Frequency in hDRG Neurons

**Fig. 4:**
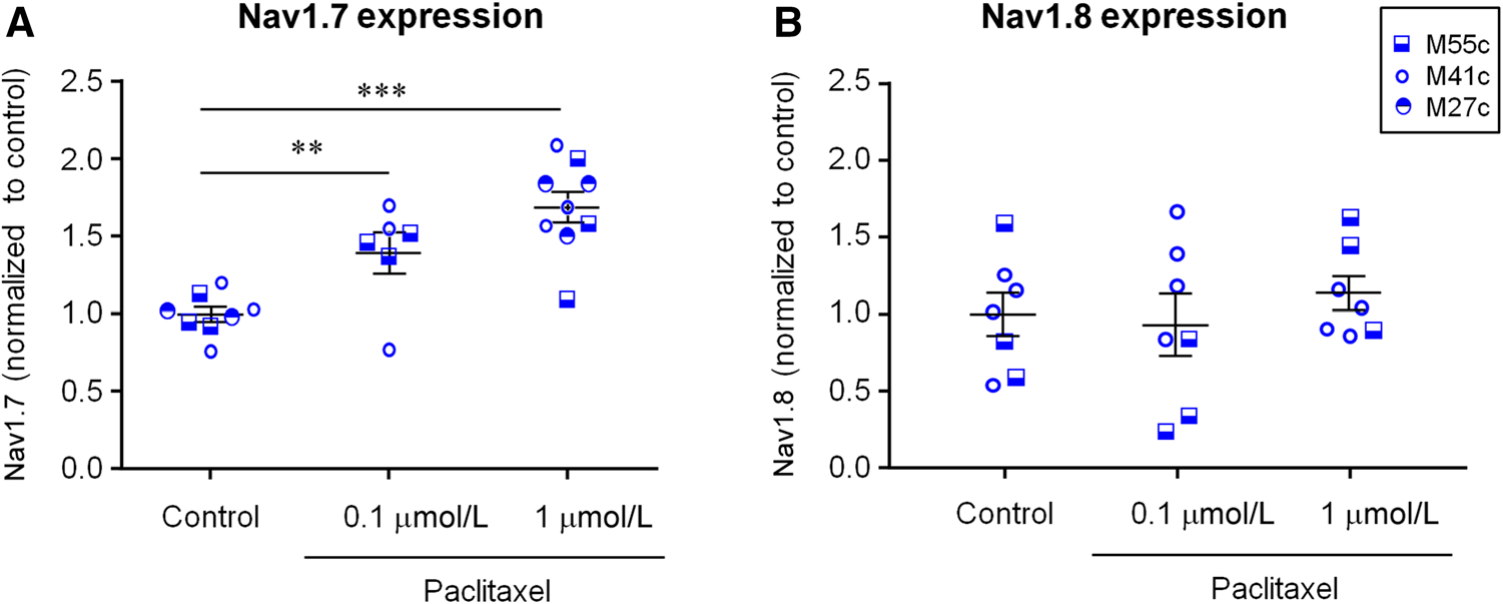
Paclitaxel increases Nav1.7 but not Nav1.8 expression in human DRGs. **A**, **B** qPCR showing Nav1.7 mRNA (**A**) and Nav1.8 mRNA (**B**) levels in hDRGs 24 h after paclitaxel treatment (0.1 and 1 μmol/L). **P < 0.01; ***P < 0.001, one-way ANOVA, *n* = 6–9 cultures/group. All the data are expressed as mean ± SEM.

Previous studies have shown that Nav1.7 is upregulated in DRG neurons under chronic pain conditions such as diabetic neuropathy, and contributes to the pathogenesis of neuropathic pain [18, 29, 30]. Paclitaxel is a widely-used chemotherapeutic drug that induces neuropathy and neuropathic pain [10, 31]. To mimic chronic pain in a dish, we treated dissociated hDRG neurons with paclitaxel (0.1 and 1 μmol/L) for 24 h. qPCR analysis revealed a significant and dose-dependent increase in Nav1.7 mRNA expression after the treatment (Fig. 4A). By contrast, Nav1.8 mRNA expression in hDRG cultures did not change after this treatment (Fig. 4B).

**Fig. 5:**
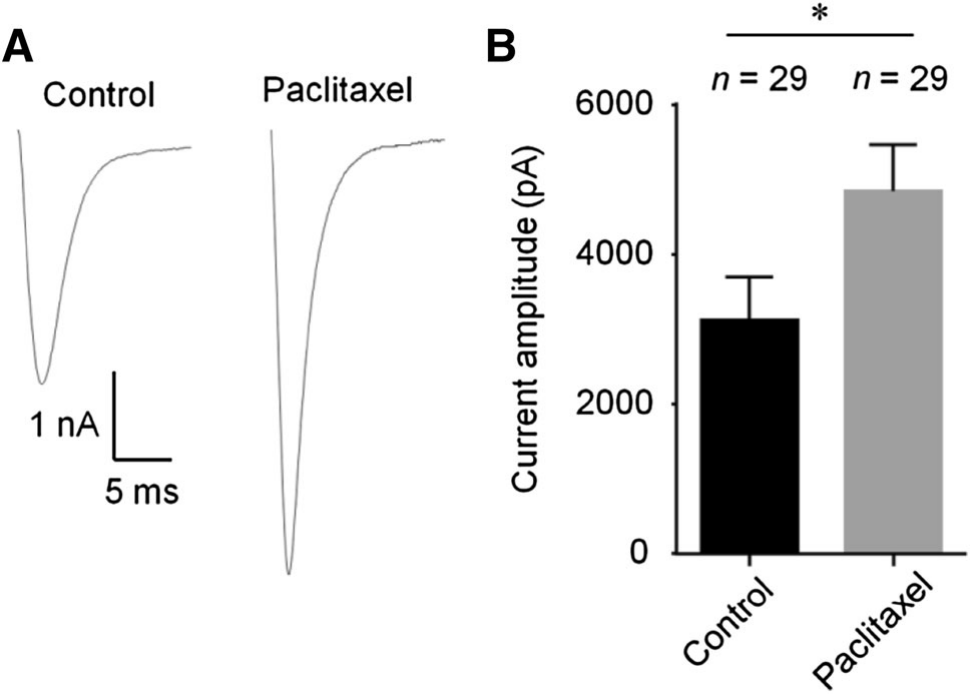
Paclitaxel increases transient sodium currents in dissociated human DRG neurons. **(A)** Traces of transient Na^+^ currents in small hDRG neurons with diameters <50 μm. Note that the current was greater after paclitaxel treatment (1 μmol/L, 24 h). **(B)** Quantification of the Na^+^ current amplitude. *P < 0.05, unpaired Student’s *t*-test, *n* = 29 neurons from 3 DRGs/3 donors. All data are expressed as mean ± SEM.

To assess the function of Navs including Nav1.7 in hDRG neurons, we recorded transient Na^+^ currents in small-diameter neurons (<50 μm). In untreated hDRG neurons, the amplitude of transient Na^+^ currents was 3131 ± 573.1 pA (*n* = 29 neurons from 3 DRGs/3 donors, Fig. 5A, B). Notably, paclitaxel (1 μmol/L, 24 h) caused a significant increase in the amplitude of transient Na^+^ currents (4853 ± 634.4 pA: Fig. 5B).

**Fig. 6:**
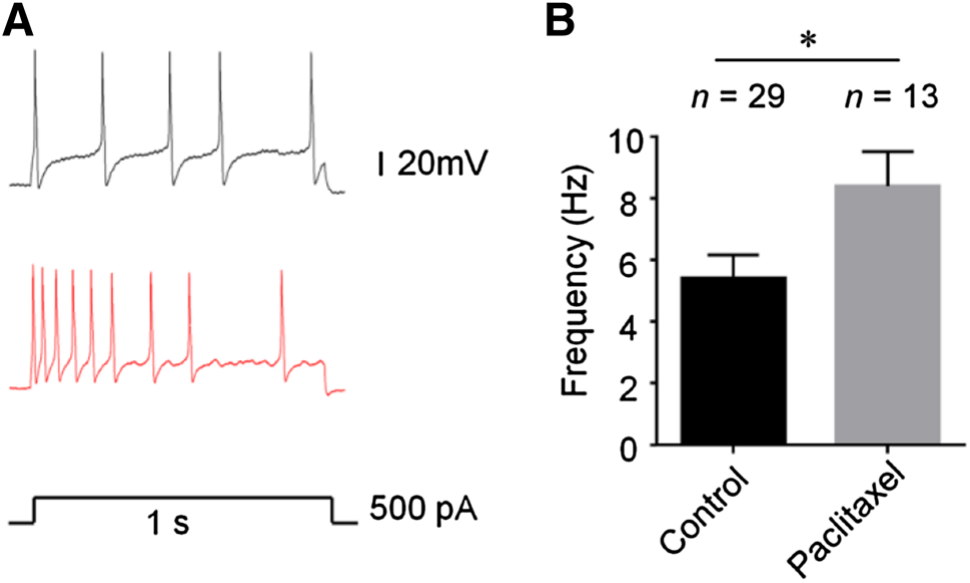
Paclitaxel increases the action potential firing frequency in human DRG neurons. **A** Traces of action potentials in small hDRG neurons with diameters <50 μm. Note that the firing frequency increased after paclitaxel treatment (1 μmol/L, 24 h). **B** Quantification of the firing frequency of action potentials. *P < 0.05, unpaired Student’s *t*-test, *n* = 13 and 29 neurons/group, from 3 DRGs/3 donors. All data are expressed as mean ± SEM.

Finally, we investigated whether paclitaxel changes the excitability of human nociceptors by recording action potentials in small hDRG neurons. The rheobase for inducing action potentials was 285.7 ± 59.5 pA (*n* = 7 neurons) in untreated hDRG neurons. After paclitaxel treatment (1 μmol/L, 24 h), there was a slight decrease in rheobase (216.7 ± 66.67 pA, *n* = 3 neurons, *P* > 0.05, unpaired two-tailed Student’s *t*-test). This lack of significance may result from the small number of neurons recorded. Current-clamp recording revealed that current injection (500 pA, 1 s) evoked action potentials in hDRG neurons (Fig. 6A). In untreated hDRG neurons, the firing frequency was 5.41 ± 0.74 Hz (Fig. 6B). Notably, paclitaxel (1 μmol/L, 24 h) caused a significant increase in firing frequency (8.71 ± 1.10 Hz: Fig. 6B). This result suggests a possible increase in the excitability of hDRG neurons after paclitaxel treatment. However, additional features of neuronal excitability such as resting potential, membrane resistance, and threshold current need to be tested in future studies.

## Discussion

There have been many failures in developing new analgesics, because (1) molecular mechanisms identified in rodents may not apply to humans and (2) animal models of pain may not represent human pains [5, 6]. To fill in this translational gap, several groups including our own have begun to test pain mechanisms in native hDRG neurons obtained from human donors [7–11]. These studies reveal that peripheral mechanisms of pain are likely to be shared by mice and humans and demonstrate a translational research strategy to improve the preclinical validation of novel analgesics. We have recently demonstrated that the autism gene *SHANK3* is expressed by both mouse and human DRG neurons and regulates TRPV1 function in the DRG neurons of both species, offering a novel peripheral mechanism of pain dysregulation in autism [11].

Although the role of human Nav1.7 has been well-studied in heterologous cells and rodent DRG neurons by over-expression of different human Nav1.7 mutants in these cells, the specific role of Nav1.7 in native hDRG neurons is unclear. Global *Nav1.7*-knockout mice recapitulate the phenotype of human congenital indifference to pain [32]. Nerve trauma after diabetes and spared nerve injury have been shown to cause upregulation of Nav1.7 in mDRG neurons and redistribution of Nav1.7 channels toward peripheral axons, leading to enhanced neuropathic pain in mice [29, 30]. However, it has also been shown that pain induced by the chemotherapeutic agent oxaliplatin and cancer-induced bone pain may not require the presence of Nav1.7 channels [33]. Furthermore, endogenous opioids appear to contribute to pain insensitivity in mice lacking Nav1.7 [34]. Thus, there could be compensatory regulations in mice lacking Nav1.7, emphasizing the importance of studying Nav1.7 in native hDRG neurons.

Our data have shown striking species differences in the expression of different Nav subtypes. Especially, the ratio of Nav1.7 expression in the hDRG (50%) is much higher than in the mDRG (20%). On the other hand, the mDRG has a much higher ratio of Nav1.8 (45%) than the hDRG (15%), and a critical role of Nav1.8 in the pathogenesis of neuropathic pain has been well documented in rodents [35, 36]. It is reasonable to postulate that the high ratio of expression of Nav1.7 in hDRG neurons is associated with an enhanced role of this subtype in human pain conditions compared with other subtypes. This notion is supported by human genetics showing that both most pain-related gain-of-function and loss-of-function mutations are found in *SCN9A* [21–23], while fewer mutations have been found in *SCN10A* (encoding Nav1.8) [8], *SCN11A* (encoding Nav1.9) [37], and *SCN8A* (encoding Nav1.6) [38]. Inherited erythromelalgia (IEM), a severe pain syndrome characterized by episodes of intense burning pain triggered by warmth, is caused by mutations in Nav1.7 in sensory and sympathetic neurons. A newly-identified IEM mutation (Nav1.7-A1632G) in a multi-generation family is responsible for hyperexcitability in sensory neurons, and the increased responses to temperature also suggest a cellular basis for the warmth-triggered pain in IEM [39]. It is noteworthy that an hDRG neuron survived for 76 years in our oldest donor (Table 1), whereas a rodent DRG neuron or a heterologous cell only survives for a few days to a few months. Thus, Nav1.7 in native hDRG neurons could be regulated by different mechanisms. Interestingly, we did not find sex- and age-dependent changes in Nav1.7 expression in hDRG neurons, suggesting that maintaining homeostasis of its expression is important for this critical ion channel. However, paclitaxel, which is known to induce neuropathy, caused a marked increase in Nav1.7 expression, associated with increases in the transient Na^+^ current and excitability (as revealed by increased action potential firing), in small-diameter (presumably nociceptive) hDRG neurons. Although other Nav subtypes could also contribute to the transient Na^+^ current, Nav1.7 is a major contributor to this TTX-sensitive current [18, 32].

In summary, naïve hDRG neurons provide a translational model in which to study voltage-gated Na^+^ channels such as Nav1.7 and “human pain in a dish”. hDRG neurons have been used to study acute inflammatory pain mechanisms following stimulation by inflammatory mediators such as prostaglandin E2 [7], but they can also be used to investigate neuropathic pain mechanisms after paclitaxel treatment as demonstrated in this study. In future studies, we will try to collect hDRGs from donors with chronic pain conditions to investigate the regulation of Nav subtypes in human pain. We will also conduct single-cell analysis to characterize the expression of Nav subtypes in different populations of hDRG neurons, as shown in mDRG neurons [40, 41]. It is noteworthy that paclitaxel did not change Nav1.8 expression in hDRG neurons, in support of a previous report that Nav1.8 does not contribute to chemotherapy-induced mechanical allodynia in rats [42]. Thus, naive hDRG neurons can be used to test new pain therapeutics such as Nav1.7 inhibitors, as the last-step of target validation before the initiation of clinical trials.
